# Testing a peer support intervention for people with type 2 diabetes: a pilot for a randomised controlled trial

**DOI:** 10.1186/1471-2296-14-5

**Published:** 2013-01-08

**Authors:** David Simmons, Simon Cohn, Christopher Bunn, Kym Birch, Sarah Donald, Charlotte Paddison, Candice Ward, Peter Robins, A Toby Prevost, Jonathan Graffy

**Affiliations:** 1 Institute of Metabolic Science, Cambridge University Hospitals NHS Foundation Trust, Cambridge, UK; 2 Primary Care Unit, Department of Public Health and Primary Care, University of Cambridge, Cambridge, UK; 3 King’s College London, Department of Primary Care and Public Health Sciences, London, UK; 4 Wolfson Diabetes and Endocrinology Clinic, Institute of Metabolic Science, Cambridge University Hospitals NHS Foundation Trust, Adden brookes Hospital, PO Box 281, Hills Road, Cambridge, CB2 0QQ, England, UK

**Keywords:** Diabetes, Peer support, Complex intervention, Self-management

## Abstract

**Background:**

People with Type 2 diabetes face various psycho-social, self-management and clinical care issues and evidence is mixed whether support from others with diabetes, ‘peer support’, can help. We now describe a 2 month pilot study of different peer support interventions.

**Methods:**

The intervention was informed by formative evaluation using semi-structured interviews with health professionals, community support groups and observation of diabetes education and support groups. Invitations to participate were mailed from 4 general practices and included a survey of barriers to care. Participants were randomized by practice to receive individual, group, combined (both individual and group) or no peer support. Evaluation included ethnographic observation, semi-structured interviews and questionnaires at baseline and post-intervention.

**Results:**

Of 1,101 invited, 15% expressed an interest in participating in the pilot. Sufficient numbers volunteered to become peer supporters, although 50% of these (8/16) withdrew. Those in the pilot were similar to other patients, but were less likely to feel they knew enough about diabetes (60.8% vs 44.6% p = 0.035) and less likely to be happy with the diabetes education/care to date (75.4% vs 55.4% p = 0.013). Key issues identified were the need to recruit peer supporters directly rather than through clinicians, to address participant diabetes educational needs early and the potential for group sessions to have lower participation rates than 1:1 sessions.

**Conclusions:**

Recruitment to a full trial of peer support within the existing study design is feasible with some amendments. Attendance emerged as a key issue needing close monitoring and additional intervention during the trial.

## Background

Diabetes related damage is often now preventable through better metabolic control (e.g. glucose, blood pressure, lipids), self care activities, regular review and timely intervention [1,2]. However, avoidable complications continue to occur [3], often due to personal and other barriers to implementing diabetes care and self care [4,5]. Psychological and psychosocial issues are often reported as particularly important barriers to diabetes care by patients, emphasizing difficulties that some have with the strictness of the diabetes regimen, including diet, exercise and monitoring [6]. For example, in the DAWN study, self-reported success with regimen adherence was relatively low in both Type 1 (46%) and Type 2 (39%) diabetes. Adherence was greater for self care than adopting other recommended lifestyle behaviours [7]. Action to overcome such barriers, so that people with diabetes are able to deal with the psychological, institutional, social and emotional issues that they face, is a major challenge.

A range of self management and structured educational programmes exist which emphasise empowerment and the pivotal role of the person with diabetes [8-11]. This is a central theme within the Chronic Care Model for disease management [12] and the UK Diabetes National Service Framework [13]. However, maintaining this role in managing an asymptomatic condition, on a day to day basis, with often unpleasant or obtrusive interventions, can be difficult. Different psychological interventions have been attempted to address this issue with varying success [14,15]. Peer support, involving experience sharing, mentoring and role-modeling, has also been proposed as a way of overcoming some psychosocial barriers through empathy, support and facilitating the seeking of knowledge or health care professional assistance should this be needed [16]. Because peer supporters have faced many of the same problems, and the support offered relates to the task of managing diabetes in one’s day to day life, peer support has the potential of being a practical way to address barriers which have been identified as so important in impeding successful diabetes self-management. A variety of individual and group approaches to providing peer support have been developed [8,16-18] using various methodologies that range from primarily educational programmes [19] to social support initiatives [20], with some being based in health services [21] and others rooted in local communities [22].

Previous research suggests that peer support interventions are welcomed by participants, but has not provided robust evidence for its utility across cultures [23]. Two randomized trials have suggested improved self efficacy and HbA1c from peer-led self management support within Spanish-speaking communities [24] and increased physical activity from peer support amongst African-American women [25]. Other trials have been conducted in Dublin (evaluating group support in primary care, including an educational component) [26,27], Warwick (evaluating telecare by peers) [28] and Michigan (comparing peer support with nurse care management) [20].

A series of large cross-cultural and international studies have begun to test the efficacy of peer support as a component of diabetes care across 8 sites, supported by Peers for Progress, a program of the American Academy of Family Physicians Foundation [29]. This research aims to specifically examine a community-based approach to peer support, rather than lay-led diabetes education. The present paper reports on the lessons learnt from a pilot study conducted in Cambridge, UK as part of the Peers for Progress initiative. The aims of the pilot were to test the feasibility and acceptability of the individual and group support programmes planned, and also to test the procedures to recruit trial participants (both as peer supporters and as peers in the programme).

## Methods

### Development of the intervention

The processes and content of the intervention were constructed from two strands of enquiry. Firstly, a discourse analysis of the literature on peer support in diabetes and other chronic conditions. Secondly, a formative evaluation based on interviews with health professionals, community activists and diabetes patients as well as observation of local diabetes education programs.

### Pilot study design

In this randomized controlled pilot study, patients registered with four general practices were recruited and each practice was randomly allocated to one of four study arms: a group-based peer support program; one-to-one support; a combination of both group and one-to-one support; with the fourth acting as a control group. In each group, support to participants (known as peers) was facilitated by peer supporters and intended to be non-directive, but operating within the trial framework.

Randomization was carried out electronically by the research team’s statistician who had no day-to-day involvement with the trial’s administration. The randomization remained unknown to the study team until after participants were recruited and the peer support sessions required co-ordination and observation.

### Recruitment

The general practices searched their registers for people with Type 2 diabetes and excluded those who were known to have Type 1 diabetes, dementia, psychotic illness or to be unsafe to visit at home. The study team did not have access to the personal data from within the practices unless provided by the peers themselves.

Potential peers were mailed three sequential invitations to join the study. A questionnaire was included within these initial letters, collecting demographic and diabetes related data as well as a ‘barriers to diabetes care’ survey [30]. The letter requested that those who were not interested in participating in the peer support research still return this survey. This survey allowed a comparison of the clinical and self reported barriers or facilitators to care among those volunteering to join the study and other survey responders.The three invitations, with a set time in between were used to maximise response. The aim was to have 8–12 peers in each intervention group based upon experience in some diabetes educational programmes [10].

After completed replies were received from peers, the study team arranged measurement sessions at the 4 general practices to which both peers and peer supporters were invited. The invitations to these sessions included a second questionnaire, which peers were requested to fill in and bring to their measures appointments. At these sessions, the research nurse obtained consent to enter the study, measured weight, height, waist circumference, blood pressure and collected blood samples (HbA1c, lipids) using standardised methodology.

### Recruiting peer supporters

The GPs and practice nurses were asked to identify a list of 4–6 people from the practice search, that they felt would be make good peer supporters using the following criteria:

Basic Knowledge – the level of knowledge of a patient who is on top of their diabetes.

People who you would get on well with – so people enjoying the contact, liking people, personable.

Flexibility, adaptability, non-judgemental – they like to be problem solvers, to connect people to answers.

Sensible approach – know what they don’t know and are comfortable with this. (This links with being responsible).

Have had Type 2 diabetes for at least 1 year

Once contacted by GPs, peer supporters were visited by a research nurse who discussed the study with them, and if both thought it appropriate that they should enter the study, completed a consent form and 2 baseline questionnaires. Potential peer supporters were also asked to complete an enhanced Criminal Record Bureau (CRB) check (as required by the ethics committee). Peers were not offered any remuneration or incentives beyond out of pocket expenses.

Following recruitment, peer supporters completed a 2.5 day training course. This included: an introduction to the trial; a session exploring the role of the peer supporter; a series of role-plays to practice boundary-setting, effective listening, dealing with difficult situations such as depression or alcoholism, and the limitations of the role (i.e. not offering knowledge or diagnosing problems); and strategies to overcome personal barriers to diabetes care. The training emphasised that the role of the peer supporter was not to replace health care professionals, but to help signpost peers to advice, services or community activities that might help them.

### Education session

To ensure that the trial assessed peer support, rather than education delivered during the course of the study, all participants were invited to a specially-designed diabetes education session, delivered by dieticians and research nurses in the study team which lasted 3.5 hours to address any important gaps in knowledge. The syllabus covered four main topics titled: ‘identifying carbohydrates and understanding portions’; ‘truths and myths about diabetes’; ‘know your numbers and medications’; and ‘keeping active and looking after your feet’. As all participants had previously diagnosed Type 2 diabetes, all had access to prior education through a local structured education programme and/or their practice nurse and/or their hospital service.

This also served to introduce the peers and peer supporters to one another. At the end of the session peers and peer supporters were asked to arrange their first peer support meetings. The peer supporters were provided with mobile phones to facilitate these contacts. This also allowed them to keep their personal contact details private. The control group attended an education session but no arrangements for peer support were made.

### Intervention

#### Meetings with peers

Peer supporters and peers were initially recruited to the intervention for 2 months. All three intervention arms were asked to meet twice during this period to discuss the practical issues arising from living with diabetes, the social and emotional challenges and the links to clinical care they use. Peer supporters were asked to deploy the listening skills explored during the peer support training to engage with their peers on these three levels and to support them in their efforts to attain better control over their diabetes and its multiple everyday consequences.

Peer supporters were encouraged to find venues in which to hold the intervention sessions in their local areas. They were offered support in this task by the study team. The study team also provided refreshments for group meetings. Peer supporters were advised that 1:1 meetings should last up to 1 hour and that group meetings should last up to 1.5 hours. The peer supporter in the combined intervention arm was encouraged to offer 1:1 and/or group peer support to peers, allowing them to choose between them or to participate in both.

During the intervention, peer supporters were asked to keep records of the number of telephone contacts they had with each of their peers, the number of meetings they held (and with whom), and to write brief reports on the content of these meetings. They were also provided with diaries in which they were encouraged to write reflections on their experiences of delivering the intervention.

#### Support for the peer supporters

Throughout the intervention period, peer supporters held monthly support meetings with the study team’s research nurse. During these sessions, peer supporters discussed the roles they were playing, the challenges and difficulties they were facing and reported on practical concerns (such as the need for additional mobile phone credit). The research nurse also provided peer supporters with a telephone number which they could contact her on during set hours in case they had pressing concerns. Finally, a committee of diabetes patients was convened to review procedures and to provide the study team with advice should a serious or sensitive event occur.

#### Evaluation and analyses

Each stage of the intervention was observed by a social scientist using ethnographic techniques. Interviews were carried out with 6 peer supporters and 12 peers (4 from each of the intervention arms). A process evaluation questionnaire was administered at the close of the pilot study, which was after 2 months of intervention. Ethnographic field notes concerning the processes and pre-intervention procedures were collated and used to produce a process evaluation which was conducted using the Medical Research Council guidelines for evaluating complex interventions [31]. Interview data was transcribed and entered into NVivo for coding using a framework analysis technique. This dataset was also cross-referenced with field notes from the observation of the peer support sessions [32].

Demographic, diabetes and barriers to care data were entered into Excel (Microsoft) and analysed using SPSS version 17 ((SPSS Inc, Il, USA). Comparisons were made using either Chi squared tests or analysis of variance. All tests are 2 tailed with p < 0.05 indicating statistical significance. Ethics approval was received by the Cambridgeshire Research Ethics Committee and all participants gave signed informed consent.

## Results

### Response and reach

**Figure 1:**
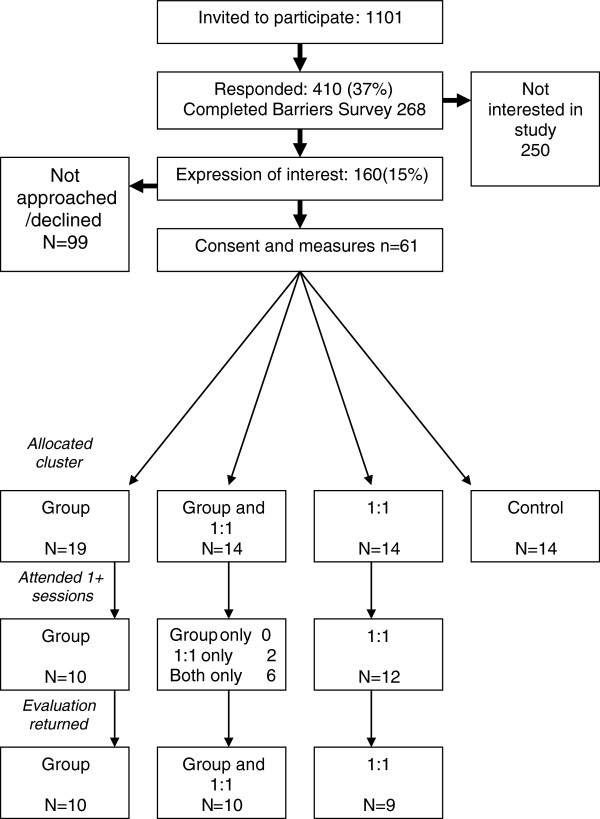
Recruitment to study.

Across the four practices, 1,101 people with Type 2 diabetes were invited to join the study. Figure 1 shows the response rate at each stage. Overall 15% expressed an interest in participating in the trial. Tables 1 and 2 show the characteristics of those completing the barriers survey overall and then compares those within the pilot and the remainder of the cohort. Those in the pilot were similar to all respondents in their demographic and clinical characteristics. Table 1 also shows the anthropometric and laboratory measures of the pilot group, who, on average were obese with reasonably well controlled HbA1c, lipids and blood pressure. Table 2 shows that those in the pilot study were significantly less likely to feel that they knew enough about their diabetes and be happy with the diabetes education/care that they had received. Otherwise, their responses were not significantly different.

**Table 1:** Characteristics of barriers survey respondents overall and by participation in pilot

	**Overall**	**Not in pilot**	**In pilot**	**sig**
N	268	207	61	
Age (years)	67.6 ± 13.2	67.6 ± 13.3	67.8 ± 13.1	ns
Male	56.4%	55.5%	59.6%	ns
Duration (years)	9.1 ± 9.3	8.8 ± 9.4	9.8 ± 9.0	ns
Glucose monitoring	55.0%	52.5%	64.3%	ns
Diabetes Tablets	65.5%	64.0%	70.9%	ns
Injects insulin	18.3%	17.9%	19.6%	ns
Blood pressure tabs	70.0%	69.2%	73.2%	ns
Cholesterol tabs	75.1%	76.0%	71.9%	ns
Smoker	9.2%	9.9%	7.0%	ns
Complications	24.8%	25.3%	23.2%	ns
Attend hospital				
For diabetes	5.5%	6.0%	3.5%	ns
HbA1c (n = 56) Mmol/mol(%)			57 ± 13(7.4 ± 1.2)	
BP(mm Hg)			137 ± 15/73 ± 10	
BMI(kg/m^2^)			31.7 ± 6.6	
Total cholesterol (mmol/l)(n = 57)			4.1 ± 0.8	
HDL cholesterol (mmol/l) (n = 57)			1.3 ± 0.3	
LDL cholesterol (mmol/l) (n = 57)			2.1 ± 0.7	

Note: characteristics are summarised using mean (SD) or percentage.

**Table 2:** Self-reported barriers/facilitators to diabetes care overall and by participation in pilot

	**Overall**	**Not in pilot**	**In pilot**	**sig**
N	268	207	61	
Knows enough about DM	57.3%	60.8%	44.6%	0.035
Knows their diabetes team	62.3%	61.6%	64.9%	ns
Other health issues impact	27.4%	27.1%	28.6%	ns
Happy with medications	80.6%	82.5%	73.6%	ns
Can afford diabetes	58.4%	58.4%	58.5%	ns
Can get to diabetes team	81.1%	80.7%	82.5%	ns
Has all DM services needed	68.2%	70.1%	61.4%	ns
DM service organisation OK	75.3%	75.2%	75.4%	ns
Pressure not to adhere	5.4%	5.9%	3.5%	ns
Others need to know more	33.5%	32.5%	36.8%	ns
Others hold DM against you	3.9%	5.0%	0%	ns
Family helping you	68.0%	66.8%	72.2%	ns
Family demands impact	5.2%	5.1%	5.5%	ns
Enough community support	58.7%	59.6%	55.6%	ns
Can always speak to the team	73.5%	74.7%	69.1%	ns
Comfortable talking with team	85.4%	84.3%	89.3%	ns
Towards diabetes care	12.1%	14.1%	5.5%	ns
Willing to look after diabetes	97.7%	97.0%	100.0%	ns
Able to look after diabetes	83.5%	84.5%	80.0%	ns
Worse symptoms	62.7%	63.3%	60.7%	ns
Enough time to look after	82.1%	81.1%	85.7%	ns
Diabetes team enough time	64.4%	65.7%	60.0%	ns
Worried/ashamed of DM	13.2%	13.8%	10.9%	ns
Willing to look after DM from today	83.3%	83.2%	83.3%	ns
Unhappy with DM team	5.9%	7.1%	1.8%	ns
Happy with education/care	70.9%	75.4%	55.4%	.013
Prefer services to be closer	13.1%	12.1%	16.4%	ns
Nothing more important than diabetes	28.4%	28.5%	27.9%	ns

### Process evaluation

Overall, 16 peer supporters agreed to participate and completed governance procedures. Of these, 8 withdrew before, and 2 during the training. Reasons cited included concerns about references to managing mental health problems, concerns about the time that might be required and having other health problems. The remaining 6 all delivered peer support interventions and completed the study.

The process evaluation identified three key areas in which the intervention could be improved: the selection and ‘clearing’ of the peer supporters; the timing and framing of the education sessions; and the role and perception of the peer supporters delivering the intervention.

#### Selecting and ‘clearing’ peer supporters

The processes through which peer supporters were selected, trained and deemed appropriate for the intervention had two unforeseen consequences. Firstly, the involvement of General Practice staff in selecting the peer supporters was significant. One peer supporter, for example, was advised by the health professional that he join the study because he was her ‘star patient’. Another peer supporter was convinced to participate because he was told by the recruiting professional that she thought he ‘would be good at it’ because of his ‘life experience’. The encouragement and validation peer supporters received from recruiting health care professionals helped capture and sustain their interest in and commitment to the study. Such messages, however, also contributed to the formation of a sense of expertise amongst some of the peer supporters. For example, during one session observed by CB, a peer supporter suggested that ‘my doctor thinks I’m quite good at looking after myself, so I’m going to try and help you too’.

A second peer supporter recruitment issue was identified during the process evaluation. As described above, peer supporters were asked to complete a CRB check, occupational health check and to sign an honorary contract. Whilst much of this process was required of us by the host institution for indemnity purposes, it was found to contribute to the sense of importance and expertise mentioned above. For example, during the final peer supporter training session, at which the peer supporters completed this process by signing the honorary contract, a conversation was captured by CB in which a peer supporter declared ‘I never thought I’d be workin’ for the hospital - ‘I’ve gone up in the world!’ Such sentiments also appeared within some of the peer support sessions. In one instance, a peer supporter introduced themselves as ‘an experienced diabetes patient, who has been asked to work with the hospital to help people like you’. In moments such as these, the formal ties the intervention provided to the institution were invoked by peer supporters as a means to stabilise their social role and construct a ‘working consensus’ with participants [33]. However, not all peer supporters displayed this tendency to cast themselves as experts or institutionally-mandated actors. Indeed, one peer supporter developed a technique for introducing the intervention and the role he was being asked to play: at the start of each session, he would say ‘I am just an ordinary person with diabetes, like you, who is here to help us all share experiences and information’. In contrast to the examples above, this peer emphasised commonality and equality in peer support sessions, and left the institutional setting ‘backstage’ [33].

#### The importance of appropriate training and education

The second set of issues identified through the process evaluation relate to the training and education provided to peer supporters and participants. The decision to train peer supporters to deliver the intervention before they received the education session with other participants proved to be unpopular. The peer supporters made it clear during training sessions, meetings with the research nurse and interviews, that they felt that the basic diabetes education should precede peer support training. One peer commented during the first day of the training session that ‘we need to know more about diabetes if we’re to support people’, ‘specially if it’s [the treatment] different to ours’. The importance of diabetes knowledge and education to those involved in the study emerged subsequently in the results to the barriers survey.

In addition to this, the peer supporters also suggested that the training relied too much on role play. For example, one peer supporter noted that during the role plays another of the peers always looked uncomfortable and awkward and felt that he did not know how to ‘play act’. Another supporter suggested that the role play seemed to make the training ‘a bit too emotional’. During a focus group with the peer supporters, facilitated by the investigators, this issue was pursued further. A further peer supporter suggested that the role play was good, as it had helped them to think about how to introduce themselves to their peers. However, the group felt that some of the role play should be substituted for ‘examples’ or ‘hypothetical problems’ that could be read out and discussed during training sessions.

The final point identified here, relates to the framing of the education sessions. The content was well received by most patients, although some felt it contained too much information to absorb in a half day session. However, observation and interview data suggest that not all participants understood how the education related to the peer support: some thought that it was the start of the peer support; others that it was a service provided by their GP practice.

#### The role and perception of peer supporters

The final cluster of issues pinpointed in the process evaluation related to the role and perception of the peer supporters. The expert status claimed by some of the peer supporters, combined with the difficulty some had distinguishing the education and support components, also suggested a degree of confusion surrounding the function peer supporters were fulfilling. This was further confirmed by observations from two separate peer support sessions. In one group session, the peer supporter ran the meeting as if it were a committee in session: the group sat around a conference table, an agenda was circulated along with a bundle of information taken from the internet, relating to the low GI diet, blood glucose testing and different kinds of insulin and minutes were taken by another group member. Similarly, another peer was discovered to have requested that a 1:1 participant demand a blood glucose monitor from their diabetes specialist nurse. Such interactions created temporary moments of disagreement between peer supporters and participants predicated on contrasting perceptions of the purpose of peer support. Whereas these two peer supporters understood their role to be one of information provision, quasi-expertise and practical involvement, the participants they worked with often saw such practices as ‘missing the point’, as one put it.

However, such interactions were exceptional, and not observed or reported across the intervention. By contrast, most of the peer supporters provided support sessions that followed the intervention guidelines. One supporter, for example, centred his group sessions on the sharing of stories. In one, he listened as a participant told the group that he travels regularly with a snooker club to play in international tournaments, which he felt kept him fit. Later in the meeting, another participant told the group that she found it difficult to know what to do when eating out. The peer supporter returned to the travelling snooker player and asked him how he managed his diet when he takes trips. Through such interactions, the peer supporter defined his role as one of facilitation and not leadership; acting to keep discussion moving and participants involved and not to impart instructions or knowledge. This was, as indicated above, the intent of the intervention design.

That two of the peer supporters interpreted the role in a more directive manner suggests that the intervention procedures did not always convey and instil the relational, supportive practices in the manner intended. These examples also reveal a key difference between the dynamics of group and 1:1 sessions. In the group setting, peer supporters were able to draw on the experiences and narratives shared by others, as the case of the snooker player details above. By contrast, the 1:1 interactions often mimicked the patient-health care professional interaction or, in some cases, a counselling style.

### Attendance at peer support

Although this was only a 2 month pilot, attendance data suggest better attendance at the 1:1 sessions (20/28, 71%, attending at least one session) than the group sessions (16/33, 48%, attending at least one session). Arranging times when all peers could attend was often difficult.

## Discussion

The pilot demonstrated that the process for recruiting into the trial would likely be sufficient for use in the main trial. The key issues identified revolved around the implementation of the trial, particularly addressing the perceived diabetes knowledge gap, the selection and training of the peer supporters and potentially peer support attendance in the group settings. In response to the findings reported above, the study investigators made a number of changes to the intervention design and study procedures.

### Modifications to the trial design

The messages of approval peer supporters received from practice staff contributed to a construction of an expert role amongst some of them. To avoid this in the future, the study team decided to alter the selection process for peer supporters for the full RCT. General Practice staff will no longer be asked to provide a short list of potential peer supporters. Rather, all those invited into the study will be given the opportunity to volunteer as peer supporters. A reference from peer supporter GPs will still be sought, but this will be considered alongside other forms of assessment.

Given that the intervention seeks to engender empowering relationships of mutual support, and not to produce ‘expert patients’, the RAPSID investigators felt that the procedures through which peer supporters were ‘cleared’ and made institutionally legitimate needed to be framed with greater care. Specifically, with the approval of the host institution, the honorary contracts have been shortened and the occupational health process scaled back, to cut the time peer supporters spend thinking about themselves as members of the hospital. Furthermore, a Standard Operating Procedure has been developed to guide the interactions in which the honorary contract is discussed with and signed by the peer supporter. Study staff will emphasise that the role of the peer supporter is voluntary, should be focussed on the needs of the communities they serve, and is not concerned with providing expert advice.

Changes have also been made to the training that peer supporters will receive and the education that will be offered to all participants. Firstly, at the suggestion of the peer supporters involved in this study, role play activities will be reduced and supplemented with case-study learning and discussion. Secondly, whilst all study materials made it clear that the education session was part of the program but was not a peer support session, a message reiterated by the dietician delivering the session, it is clear that the procedures for the full RCT will need to make this more readily comprehensible. In response to these two issues, the investigators have agreed to distribute a basic diabetes educational booklet during the education session. Participants and peer supporters will be requested to refer to this booklet when they have knowledge-related questions. If information is not within the booklet, then theyare advised to ask their practice nurse. The intention is that this will reduce the feeling of being overwhelmed by information, by providing a source participants can return to, and also facilitate participants to identify the education session as a distinct part of the peer support program.

Finally, in response to the perceptions of the peer supporters that were constructed during the pilot, the investigators have made a number of alterations for the main trial. Firstly, the peer supporter role will be re-titled ‘peer support facilitator’ (PSF), to emphasise the collaborative nature of support, and minimise the perception that the support emanates only from the ‘supporter’. Secondly, PSFs will be provided with a resource booklet, which contains a number of activities and ideas for stimulating supportive discussion (along with study documentation). Finally, the PSF selection process will now also include an assessment of potential PSFs’ ability to listen and interact on a 1:1 level (during baseline measurements) as well as a group level (during the education session).

Attendance will be observed closely over the first 3 months through the allocated RAPSID nurse in the main trial. Where attendance is low, an investigation will be conducted by study research nurses to identify ways to increase attendance (if possible). The main trial will compare attendance as one of its a priori analyses.

### Connections with other diabetes peer support research

A recent randomised controlled trial of peer support based upon general practice populations in Ireland suggested that while group based peer support could be delivered in a primary care setting, it had no impact on metabolic measures or quality of life [27]. The intervention we are now testing is community based, and the changes we have made to the PSF recruitment process strengthen this connection.

A further issue that we have grappled with, was that the average HbA1c of those participating was usually within the target range. This was also found by Smith et al. [27]. With a mean HbA1c of 57 ± 13 mmol/mol, there may be room among many participants to improve their HbA1c. While the results from ACCORD trial led to reviews of the HbA1c targets in Type 2 diabetes [34], tailoring of targets has been emphasised [35] and the American Diabetes Association recommends lowing HbA1c to < 53 mmol/mol in most patients [36]. Nevertheless, the prior Quality Outcomes Framework target of 53 mmol/mol (the lower HbA1c target for pay for performance incentives for primary care) has now shifted to 58 mmol/mol, which would potentially allow patients choosing to lower their HbA1c to do so-potentially with less medication. From a study point of view, we debated the advantages and disadvantages of only including those with hyperglycaemia in the study, but felt that this would potentially undermine the benefit of mixing those patients who are able to manage their diabetes well with those who find it more difficult.

Finally, a further parallel with the Dublin study was found. The investigators noted that they had underestimated the organisational complexity of running a peer support trial [27]. As Figure 1 shows, as patients moved through the pilot study, numbers dwindled. Whilst perfectly normal for such studies, this highlights the need for careful planning and management in such interventions, especially when targeting large numbers. This finding is especially important for service architects considering implementing peer support programmes.

## Conclusion

We conclude that although recruitment into a randomised controlled trial of facilitated, non directive peer support in diabetes is feasible, it requires very careful preparation, management and an understanding of the population involved. The method for selection of the peer supporters, the style of training provided to the peer supporters, the existing level of diabetes knowledge of all participants and the need for a clear sense of how diabetes education relates to peer support, were key issues that would have impacted significantly on a full trial had the pilot not taken place.

## Competing interests

The authors declare that they have no competing interests.

## Authors’ contributions

DS developed the overall design. DS, CB, JG and ATP undertook the analyses. All co-authors contributed to the final design, drafting of the paper and have given approval for publication.

## Pre-publication history

The pre-publication history for this paper can be accessed here:

http://www.biomedcentral.com/1471-2296/14/5/prepub
